# Supplementary material for: Genome-Wide Analysis in Drosophila Reveals the Genetic Basis of Variation in Age-Specific Physical Performance and Response to ACE Inhibition
Source: Genes (Basel). 2022 Jan 14;13(1):143. doi: 10.3390/genes13010143 (PMC8775566; doi:10.3390/genes13010143)
Supplement: Supplementary file 1 [file genes-13-00143-s001.zip › Genes Final 1_13_22 supplemental files.pdf]

Supplementary Materials for

"Genome-wide analysis in Drosophila reveals the genetic basis of variation in age-specific physical performance and response to ACE inhibition"

Mariann M. Gabrawy[1,2], Nick Khosravian[1], George S. Morcos[1], Tatiana V. Morozova[3], Meagan Jezek[1], Jeremy D. Walston[2], Wen Huang[4], Peter M. Abadir[2], Jeff Leips[1]*

*Corresponding author. Email: leips@umbc.edu

This section includes:

Figure S1. Climbing speed at young age varies with genotype.
Figure S2. The effects of age on climbing speed, sensitivity, and drug response depend on genotype.
Figure S3. Endurance at young age varies with genotype.
Figure S4. The effects of age on endurance, sensitivity, and drug response depend on genotype.
Figure S5. Gene networks for young age physical performance traits.
Figure S6. Genetic networks for climbing speed and endurance combined.
Figure S7. Genetic networks of human orthologs for physical performance.
Figure S8. Genetic networks for sensitivity to Lisinopril treatment at young age.
Figure S9. Lisinopril treatment effect on endurance in RNAi genotypes.
Figure S10. Verification of *in vivo* RNAi knockdown using qRT-PCR.

Data files submitted as separate Excel files associated with this manuscript:

Supplementary Table S1. Physical performance results and means of 126 DGRP lines submitted to DGRP pipeline.
Supplementary Table S2. GWA results for physical performance.
Supplementary Table S3. Candidate gene information.
Supplementary Table S4. Sensitivity of 126 DRGP lines to Lisinopril treatment results.
Supplementary Table S5. GWA results for sensitivity to Lisinopril.
Supplementary Table S6. Physical performance of *in vivo* RNAi lines.
Supplementary Table S7. qRT-PCR of *in vivo* RNAi-*axn*, RNAi-*fz*, RNAi-*nemo*, RNAi-*wingless*

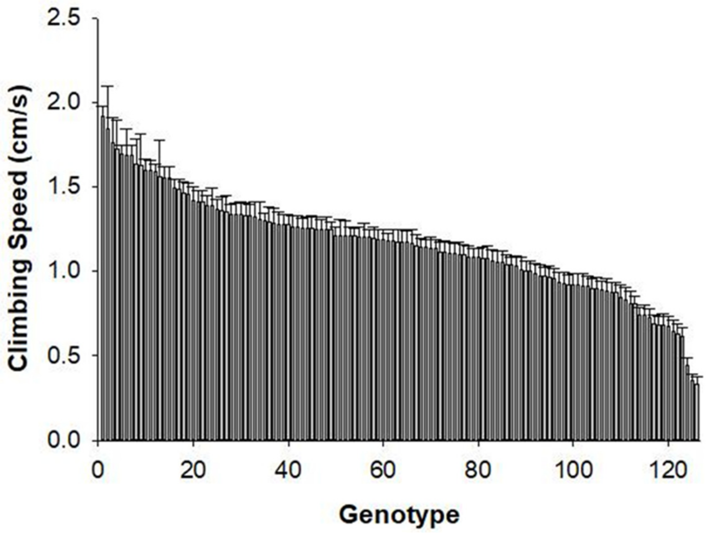

Figure S1. Climbing speed at young age varies with genotype. Data are the mean climbing speed for each DGRP line tested (± one S.E.) independent of age and treatment. Genotypes are ranked from fastest to slowest.

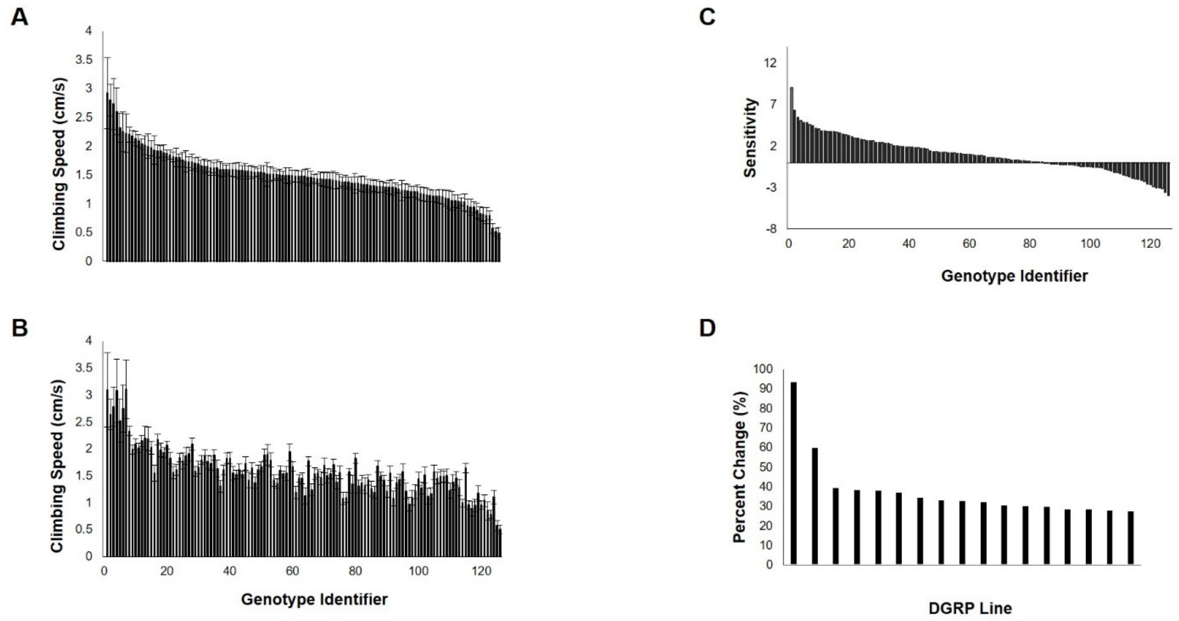

Figure S2. The effects of age on climbing speed, sensitivity, and drug response depend on genotype. (**A**) Genetic variation in climbing speed at young age; genotypes are ranked from fastest to slowest. (**B**) Genetic variation in climbing speed at old age; genotypes are ranked based on climbing speed at young age. Data are the mean climbing speeds for each DGRP line tested (± one S.E.) at each age. (**C**) Sensitivity at young age depends on genotype. There is significant variation among genotypes in the sensitivity of climbing speed to Lisinopril treatment at young age. (**D**) DGRP lines with an improved or worsened climbing speed of equal or greater than 25% when treated with Lisinopril.

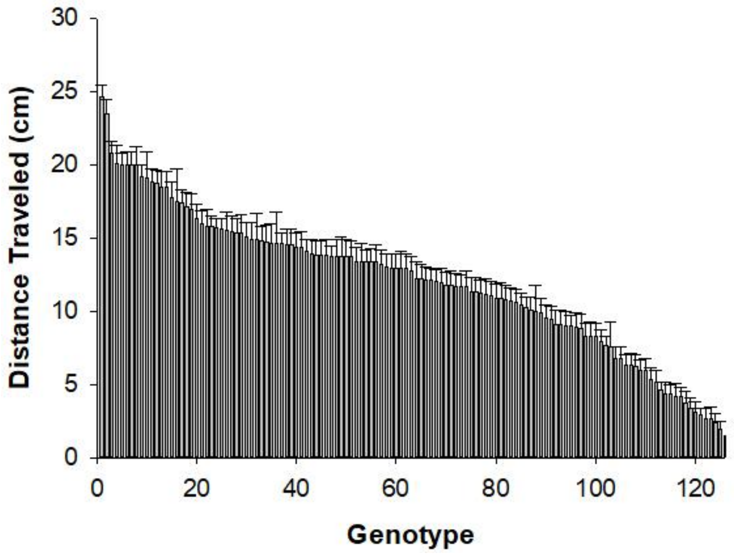

Figure S3. Endurance at young age varies with genotype. Data are the mean distance reached in 15 seconds for each DGRP line tested (± one S.E.) independent of age and treatment. Genotypes are ranked from longest to shortest distance traveled.

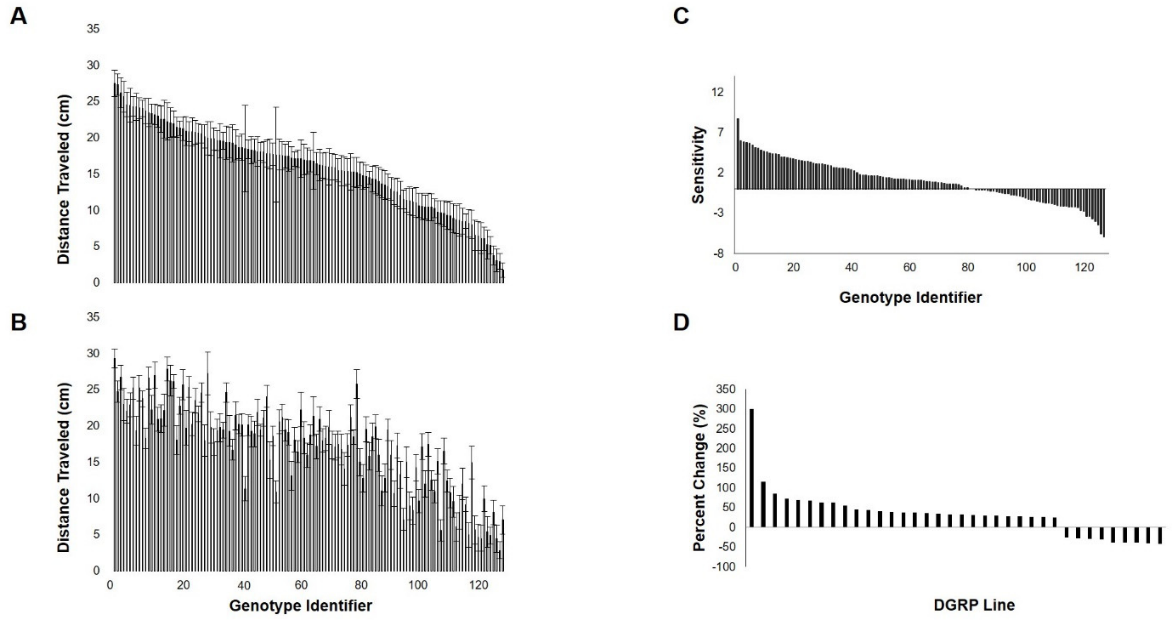

Figure S4. The effects of age on endurance, sensitivity, and drug response depends on genotype. There is a significant genotype-by-age interaction. (**A**) Variation among genotypes in endurance at young age; genotypes are ranked from longest to shortest distance reached. (**B**) Variation among genotypes in endurance at old age; genotypes are ranked from longest to shortest distance travelled at young age. Data are the distance travelled reached for each DGRP line tested (± one S.E.) at each age; ANCOVA. (**C**) Sensitivity at young age depends on genotype. There is significant variation among genotypes in the sensitivity of endurance to Lisinopril treatment at young age. (**D**) DGRP lines with an improved or worsened endurance of equal or greater than 25% when treated with Lisinopril.

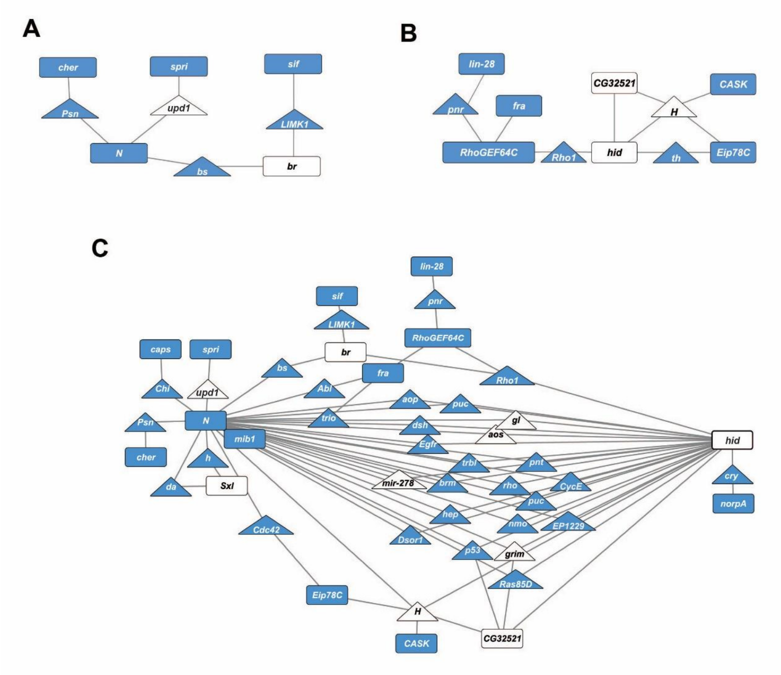

Figure S5. Genetic networks for endurance. (**A**) A genetic network for endurance at young age. (**B**) A genetic network for endurance at old age. (**C**) A genetic network for endurance combining both age groups. The networks were derived from candidate genes identified in GWA analyses for endurance, using data from flies on control and Lisinopril treated food within an age group (**A**, **B**) or using data from both ages (**C**). See legend Figure 3 for description of symbols in figure.

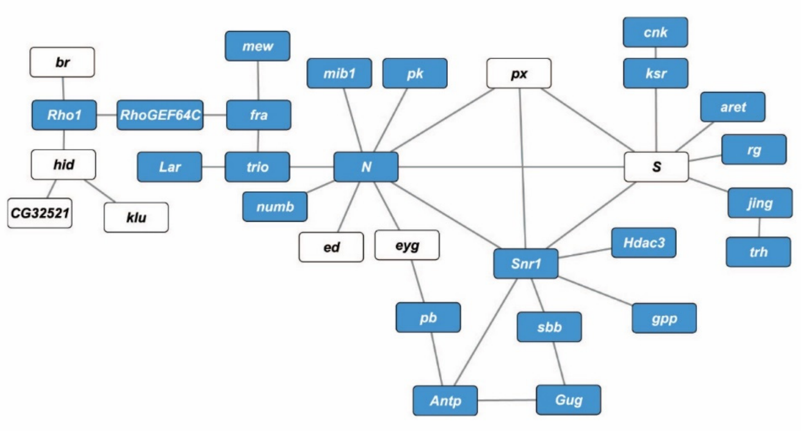

Figure S6. Genetic networks for climbing speed and endurance combined. The network was derived from candidate genes identified in GWA analyses for (**A**) climbing speed and (**B**) endurance at both ages, using data from flies on control and Lisinopril treated food. See legend Figure 3 for description of symbols in figure.

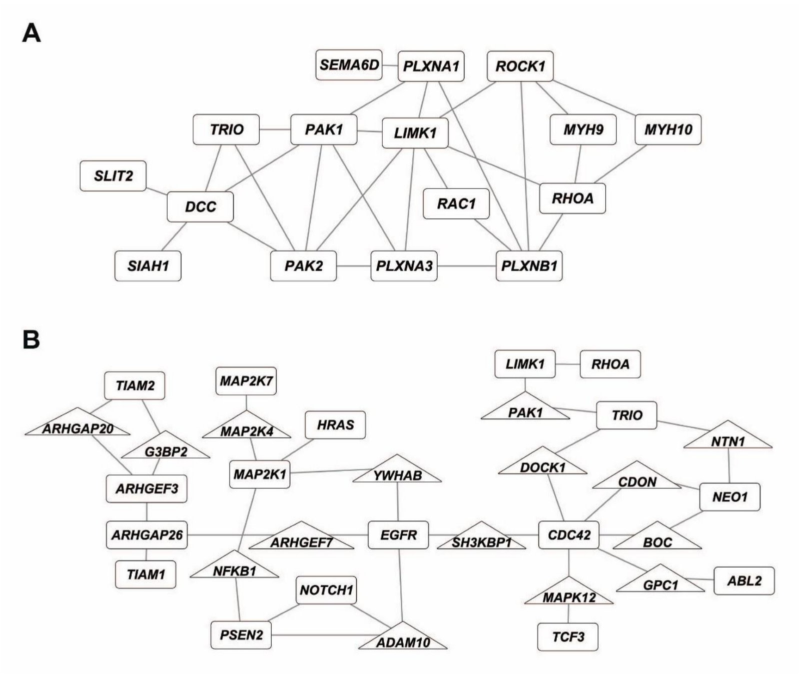

Figure S7. Genetic networks of human orthologs for physical performance. (**A**) A genetic network of human genes for climbing speed (**B**) A genetic network of human genes for endurance. The networks were derived using interactions of human orthologs of candidate genes identified in GWA analyses for climbing speed and endurance. Genes in boxes are human orthologs of genes identified in the GWA of *Drosophila*.

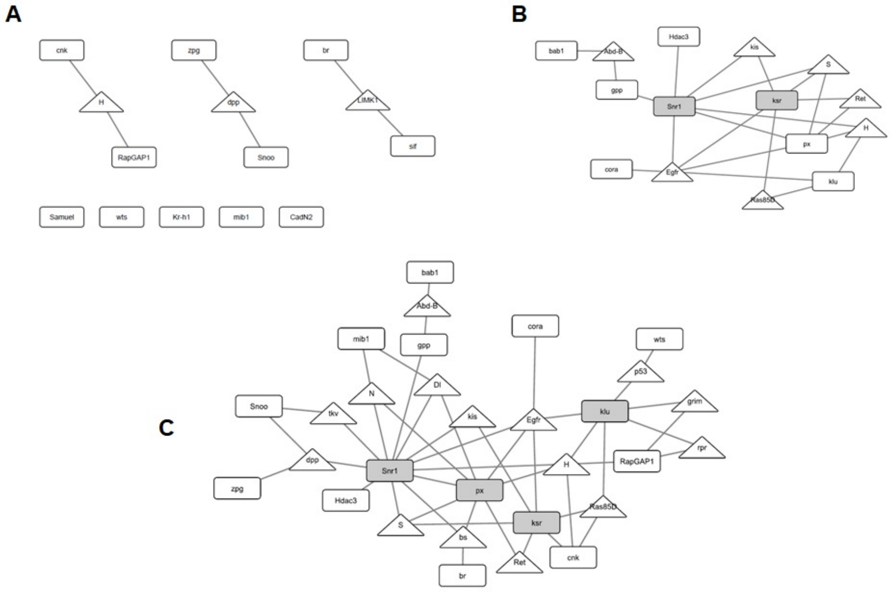

Figure S8. Genetic networks for sensitivity to Lisinopril treatment at young age. (**A**) A genetic network for climbing speed young age for control flies. (**B**) A genetic network for climbing speed at young age for Lisinopril-treated flies. (**C**) A genetic network for climbing speed at young age for sensitivity and Lisinopril-treated flies. The networks were derived from candidate genes identified in GWA analyses for climbing speed using data from flies on control and Lisinopril treated food within young age. See legend Figure 3 for description of symbols in figure.

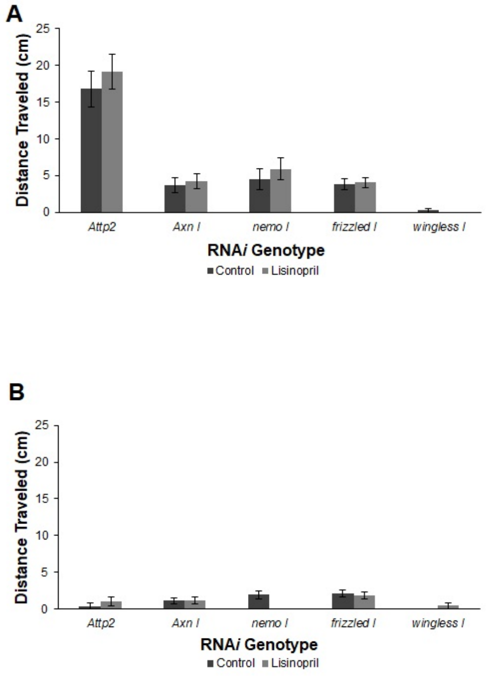

Figure S9. Lisinopril treatment effect on endurance in RNA*i* genotypes. Genetic knock down effect on endurance. (**A**) Endurance of control and Lisinopril-treated RNA*i* genotypes at young age. (**B**) Endurance of control and Lisinopril-treated RNA*i* genotypes at old age.

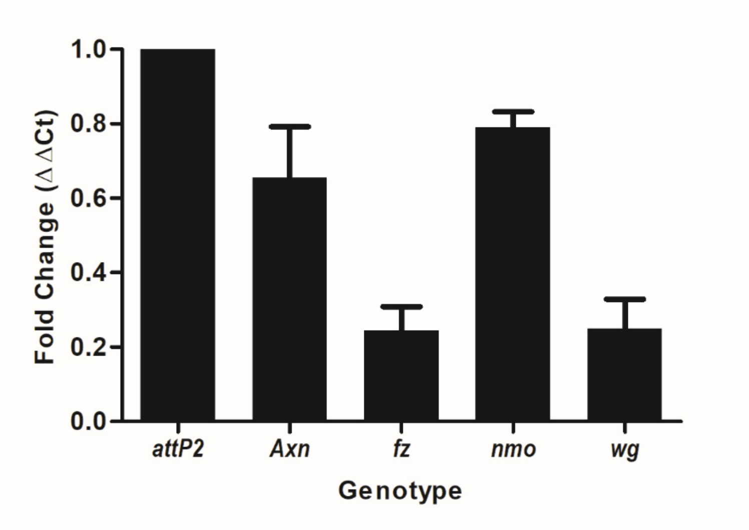

Figure S10. Verification of *in vivo* RNAi knockdown using qRT-PCR. Fold change in expression is relative to the control line of each RNAi cross. *attP2* = F_1 offspring of cross *dj667-Gal4* x *attP2*, *Axn* = F_1 offspring of cross *dj667-Gal4* x *UAS-Axn-RNAi, fz* = F_1 offspring of cross *dj667-Gal4* x *UAS-fz-RNAi, nmo* = F_1 offspring of cross *dj667-Gal4* x *UAS-nmo-RNAi, wg* = F_1 offspring of cross *dj667-Gal4* x *UAS-wg-RNAi*. S.E. bars.

Supplementary Table S1. qRT-PCR of in vivo RNAi-axn, RNAifz, RNAi-nemo, RNAi-wingless. (A) Climbing speed of control and Lisinopril-treated flies at one and old age. (B) Endurance of control and Lisinopril-treated flies at one and old age.

Supplementary Table S2. Sensitivity of 126 DRGP lines to Lisinopril treatment results. (A) Candidate SNPs associated with age-specific climbing speed at $P < 10^{-5}$. (B) Candidate SNPs associated with age-specific endurance at $P < 10^{-5}$. Output is from the DGRP Freeze 2 analysis pipeline.

Supplementary Table S3. Physical performance results andmeans of 126 DGRP lines submitted to DGRP pipeline. Candidate genes associated with each physical performance phenotype (A) climbing speed and (B) endurance.

Supplementary Table S4. GWA results for physical performance;. (A) Climbing speed calculated sensitivity at one and old age. (B) Endurance calculated sensitivity at one and old age.

Supplementary Table S5. Candidate gene information. (A) Candidate SNPs associated with age-specific sensitivity of climbing speed to Lisinopril treatment at $P < 10^{-5}$. (B) Candidate SNPs associated with age-specific sensitivity of endurance to Lisinopril treatment at $P < 10^{-5}$. Output is from the DGRP Freeze 2 analysis pipeline.

Supplementary Table S6. GWA results for sensitivity to Lisinopril. (A) Climbing results of *in vivo* RNAi-*axn*, RNAi-*fz*, RNAi-*nemo*, RNAi-*wingless* in Lisinopril-treated flies at young and old age. (B) Endurance results of *in vivo* RNAi-*axn*, RNAi-*fz*, RNAi-*nemo*, RNAi-*wingless* in Lisinopril-treated flies at young and old age.

Supplementary Table S7. Physical performance of in vivo RNAi lines.
